# Apparent Contact Angle around the Periphery of a Liquid Drop on Roughened Surfaces

**DOI:** 10.1038/s41598-020-65122-w

**Published:** 2020-05-19

**Authors:** Xuemin Huang, Ian Gates

**Affiliations:** Department of Chemical and Petroleum Engineering, University of Calgary, Calgary, Canada

**Keywords:** Fluid dynamics, Fluidics, Chemical engineering

## Abstract

The wetting of roughened surfaces is complicated since not all of the surface of the irregular surface is wetted and thus, the three-phase contact line for the liquid drop is a complex, three-dimensional line that varies according to the dimensions of the roughness and its spatial heterogeneity. This can cause the contact line to not sit within a constant height horizontal plane especially when air is trapped underneath the liquid layer. Here, we explore the effect of roughness on the effective contact angle of a water droplet on a roughened hydrophobic surface. The results show that the apparent contact angle varies around the periphery of the droplet due to the roughness of the surface on first contact. Also, repeated wetting of the droplet on the surface reveals that the apparent contact angle changes due to residual liquid remaining on the roughened surface. The results also show that the Wenzel and Cassie-Baxter models tend to overestimate the apparent contact angle on the roughened surfaces.

## Introduction

The equilibrium contact angle is a characteristic measure of the energy state at a liquid-fluid-solid contact line. The contact angle is often discussed in the literature, but when it comes to a real surface, it is questionable whether it reflects the wettability of that surface by a single contact angle measurement^1–3^. The behaviour of the contact line on a roughened surface is complex because it depends not only on the implicit wettability of the solid (which in turn depends on the compositions of solid, liquid, and fluid reflected by the surface energies of the interfaces and solid) but also the spatial properties of the roughness of the surface^4–8^. In other words, the apparent wettability of the roughened surface depends on the length scale of the roughness relative to the size of the drop as well as the physiochemical properties of the surface^5,7^. For example, the topography of the roughened surface can play a role on droplet movement on the surface: angular sharp grains or smooth rounded grains or a combination of both will affect how the contact line arranges itself on the roughened surface^5,8,9^.

In nature, there are plants and insects that have irregular rough surfaces which exhibit high hydrophobicity^10^. Inspired by these natural examples, rough surfaces have become a focus in the search for ultra-hydrophobic surfaces^2,5,11,12^. When describing the wettability of a rough surface by measuring a fluid-liquid-solid contact angle, it becomes complex because of the difficulty of thoroughly considering all variables, such as surface chemical heterogeneity, trapped air under the liquid within the roughness structure of the surface which makes the liquid only contact parts of the solid^2,5,7,13^. Gravity will reshape the liquid droplet and flatten the contact line to some extent depending on the weight of the droplet^14^. During contact angle measurement, the difference of the scales of the liquid droplet and solid grain affects contact line placement length and configuration. If the volume of droplet is small relative to the roughness, measurement may not sufficiently reflect the characteristics of the solid surface^2,7,15^. Other factors that affect how contact lines evolve on a surface include evaporation and vibration^16^. Youngblood and McCarthy^17^ demonstrated that roughness at molecule scale can significantly improve the hydrophobicity for the surfaces made of the same material. Along with the development of precision nanoscale manufacturing, surfaces can be specially manufactured at nanometer scale to obtain positive impacts of roughness on both hydrophobicity and hydrophilicity, depending on the original wettability of the surface^18^. Furthermore, results show that roughness can affect the wettability in the same manner for much larger scales^19^.

**Figure 1:**
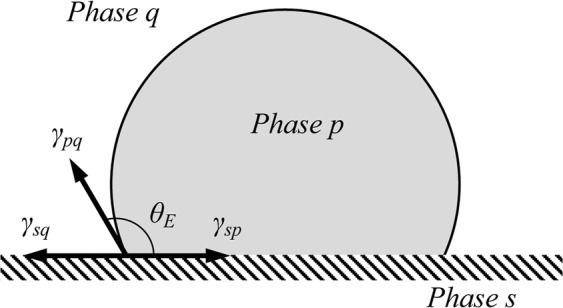
Three-phase contact line on a flat surface.

**Figure 2:**
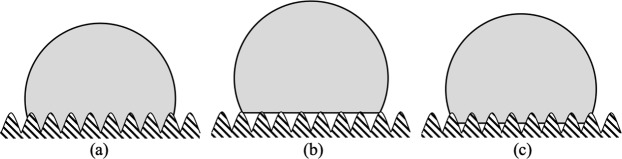
Wetting on rough surfaces: (**a**) Wenzel’s model showing liquid phase completely filling the valleys, (**b**,**c**) Cassie-Baxter’s model showing liquid phase standing on top of the solid surface peaks and liquid filling part of the valley with air is trapped below.

Figure 1 shows classic ideal wetting phenomenon of liquid phase *p* and fluid phase q on a flat solid phase *s*. The forces at the contact line between the three phases can be described by Young’s equation:^2,20,21^

1 $${\gamma }_{sp}={\gamma }_{sq}+{\gamma }_{pq}\,\cos \,{\theta }_{E}$$

where ${\gamma }_{sp}$ is the interfacial tension between solid and liquid phase $p$, ${\gamma }_{sq}$ is the interfacial tension between solid and fluid phase $q$, $\,{\theta }_{E}$ is the equilibrium contact angle, and ${\gamma }_{pq}$ is the interfacial tension between phases $p$ and $q$. Young’s equation deals with the contact of a fluid-liquid-solid system at macroscopic scale and on a smooth surface. In reality, there is no such thing as a truly smooth surface. When a liquid droplet sits on a rough surface, the measurement of ‘constant’ contact angle is meaningful only when the liquid droplet size is significantly greater than the wavelength of rough surface^2,3,22^. Two models have emerged to describe wettability on a rough surface, as reflected by an apparent contact angle: the Wenzel and Cassie-Baxter models, illustrated in Fig. 2.

### Wenzel model

As shown in Fig. 2(a), Wenzel’s approach is the case where the liquid fills the valleys of the rough surface, so that the apparent contact angle can be expressed as following^23^:

2 $$\cos \,{\theta }_{A}=R\,\cos \,{\theta }_{E}$$

where ${\theta }_{A}$ is apparent contact angle and *R* is the ratio of the actual area of contact surface to the projected area on the horizontal plane (the roughness ratio); this implies that *R* > 1 on a rough surface.

### Cassie-baxter model

Displayed in Fig. 2(b), Cassie-Baxter’s approach assumes that the liquid droplet is supported on the top of the peaks of a rough surface. The apparent contact angle, for flat topped roughness structures, can be described by^24^:

3 $$\cos \,{\theta }_{A}=Rf\,\cos \,{\theta }_{E}+f-1$$

where *f* is the solid area fraction of the liquid-solid contact area. More generally, the apparent contact angle for roughened surfaces is given by^2,5^:

4 $$\cos \,{\theta }_{A}={R}_{f}f\,\cos \,{\theta }_{E}+f-1$$

where ${R}_{f}$ is the roughness ratio of only the wetted solid.

Wenzel’s model assumes perfect contact between the liquid and the rough surface valley whereas on the other hand, Cassie-Baxter’s model introduces a separation between the liquid and valleys of the rough surface. With the introduction of a third phase (the most common being air), as shown in Fig. 2(c), the liquid partially fills the valley^25^. From a three-dimensional (3D) view, the valleys of the rough surface can be connected or isolated depending on the structure of the roughness, and coverage of the liquid on the surface, which in turn, affects the actual contact line between the fluid and liquid phases at the solid surface. Rough surfaces are unique yielding different apparent contact angles. What becomes clear from the literature is that the effect of roughness on the apparent contact angle is not clear and that the variability of the apparent contact angle associated with the scale of roughness is unknown. In this paper, we focus on the wettability of water on macroscale hydrophobic roughened surfaces to explore this and how the apparent contact angle varies along the periphery of a droplet.

## Method and Materials

Two solid materials were used to construct the roughened surfaces used in the experiments described here: natural silica sand and manmade silica glass beads. The natural silica sands was obtained from a surface mined oil sands deposit in the McMurray Formation in Northwestern Alberta. The grains are angular with diameter ranging from 100 to 200 microns with mean diameter equal to 156 microns. The glass beads are roughly spherical in shape with diameter ranging from 350 to 450 microns and mean equal 415 microns. Naturally, both the sand and glass beads are water wet. Both the sands and glass beads are treated to make them hydrophobic as described in the following.

### Preparation of hydrophobic sands

**Figure 3:**
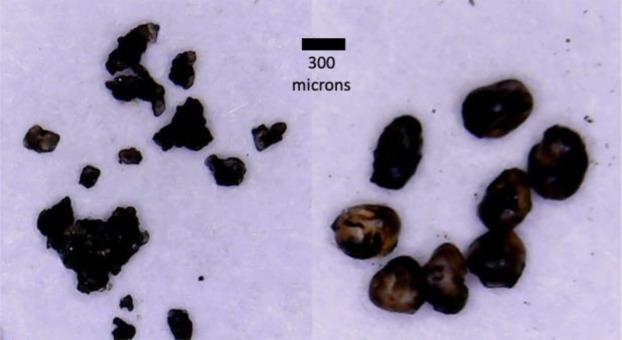
Coated sands and glass beads (left are sand grains; right are glass beads).

To obtain hydrophobic sand, in the first step, the sand were mixed gently into a beaker of heated (60 °C) bitumen until all of the grains were coated with bitumen. Next, the grains were left to sit for one day at room temperature (22 °C) open to the atmosphere. Next, toluene was mixed with the coated grains with a volume ratio of toluene-to-solids equal to 1:1. After the sand-bitumen were totally mixed with toluene, the liquid was filtered. This toluene extraction step was repeated three times. The remaining sand-oil mixture was then placed in an oven at 130 °C to evaporate the remaining toluene and light ends. After the sand was totally dried, the residual hydrocarbon evenly coated the sand grains. The same procedure was used for the glass beads. Figure 3 displays the coated sands and glass beads. For comparison with a smooth silica surface, a glass plate was also treated using the same procedure.

### Preparation of roughened layers

Three roughened layers were generated. The first consisted of sand only, the second of glass beads only, and the third was a combination of sand and beads with volume fraction two-thirds sand and one-third glass beads. The solids were placed on a glass substrate (1 cm by 1 cm) and by using a uniform gap (1 mm) operated as a knife coater dragged slowly across the solid deposited on the substrate, a smooth and uniform solid thickness layer was created on the glass substrate. Thereafter, a second glass substrate was placed on top of the sand layer with a weight (10 g) on top to slightly compress the sand grains together. After six hours, the weight and top glass substrate were removed carefully from the sand layer. Due to residual hydrocarbon coating on the grains, the sand grains were consolidated together to form a cohesive roughened surface.

### Roughness measurement

**Figure 4:**
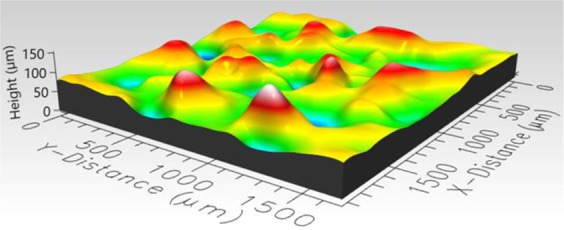
3D image of hydrophobic sand surface (maximum peak height 81.87 μm, mean valley depth 72 μm, arithmetic mean height 16.97 μm, skewness 0.1759, kurtosis 3.382).

**Figure 5:**
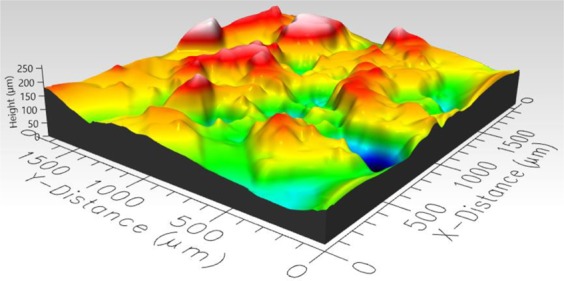
3D image of hydrophobic glass bead surface (maximum peak height 261.4 μm, mean valley depth 143.6 μm, arithmetic mean height 31.67 μm, skewness −0.05579, kurtosis 3.202).

**Figure 6:**
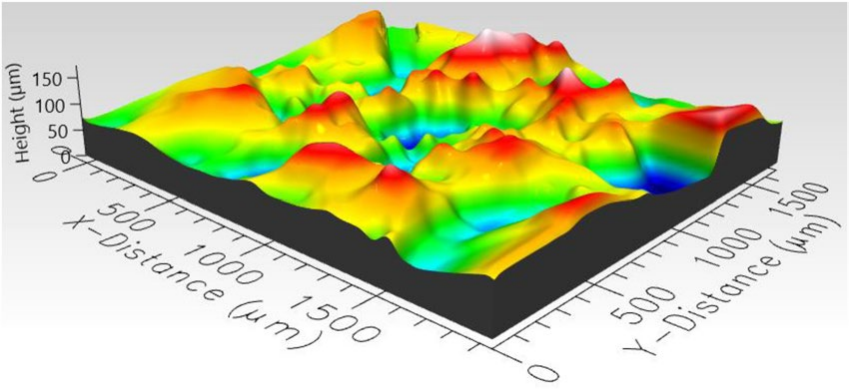
3D image of hydrophobic mixed sand-bead surface (maximum peak height 89.9 μm, mean valley depth 83.01 μm, arithmetic mean height 22.73 μm, skewness −0.1366, kurtosis 2.736).

**Figure 7:**
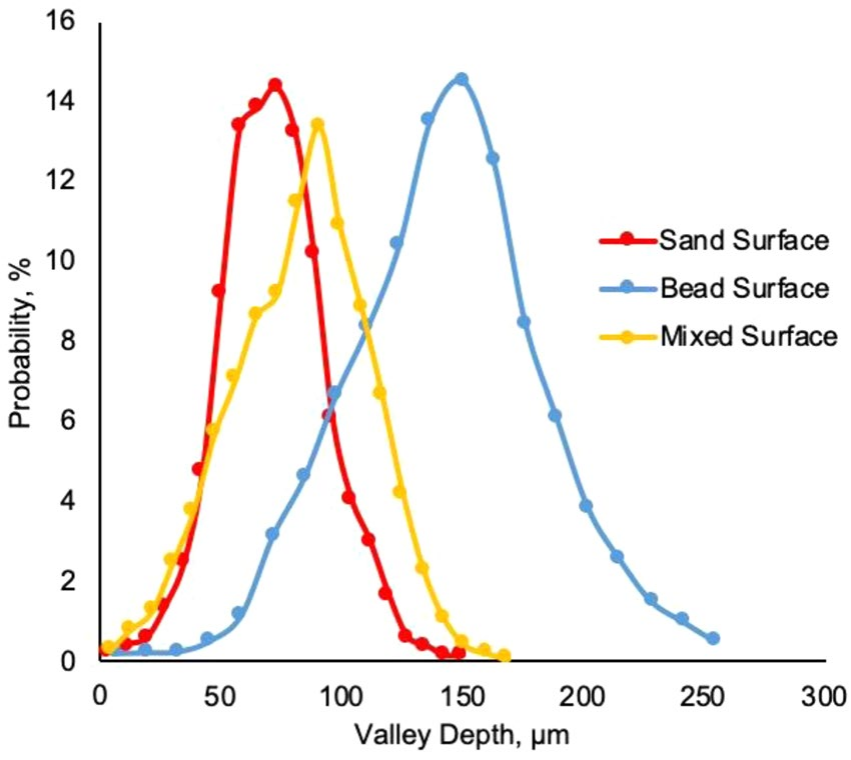
Distribution of average height of three surfaces.

To determine the roughness of the hydrophobic roughened surfaces, a three-dimensional Profilometer (Profilm3D, Filmetrics Inc.) was used to measure roughness parameters of the three surfaces. 3D views of hydrophobic sand, glass bead, and mixed sand-bead surfaces are presented in Figs. 4–6, respectively. The composition and roughness of each generated hydrophobic surface are listed in Table 1 with distributions of the heights displayed in Fig. 7. In Table 1, the surface zero represents the coated hydrophobic glass substrate, which gives the properties as a reference. The arithmetic mean height which is commonly used as a surface roughness parameter, is defined as the arithmetic mean or average of the absolute distances of the surface points from the mean plane. For a random rough surface, the mean plane is not smooth so that the average valley depth is used as well to describe the roughness of the surface. The average valley depth is defined as the average absolute height of the sample surface. For the sand, an evaluation of three separate samples of the sand layer provided an average arithmetic mean height equal to 16.97 μm, average valley depth equal to 72 μm, with maximum height equal to 81.87 μm. For the glass beads, the average values of the arithmetic mean height, average valley depth, and maximum height for three separate samples was equal to 31.67 μm, 143.6 μm, and 91.82 μm, respectively. Similarly, for the mixture of sand and bead, the values were 22.73 μm, 83.01 μm, and 89.9 μm, respectively. The distributions of the height shown in Fig. 7 show that they generally follow a normal distribution with the sand having a narrower distribution and the beads having a wider distribution.

**Table 1:** Properties of rough surfaces.

Surface	Composition of Surface	Volume fraction sands	Volume fraction glass beads	Roughness (Mean Valley Depth), µm	Roughness (Arithmetic Mean Height), µm
0	Smooth glass	0	0	<0.15	<0.15
1	Sand	1	0	72.00	16.97
2	Mixed	0.666	0.333	83.01	22.73
3	Glass Beads	0	1	143.6	31.67

### Contact angle measurement

A fixed volume syringe (Model Pipetman Neo P20N, Gilson Inc.) is used to provide each water droplet at a volume equal to 5 µL which gives each water droplet the weight of 5 mg. Distilled (DI) water was used for all experiments. For each droplet, it is placed from about <2 mm above the surface so that during the process of the droplet falling on the solid surface, the deformation of the liquid droplet due to acceleration and impact is negligible. The enclosure environment is maintained constant at 22 °C with relative humidity of 43% with no air motion. The droplet size diameter is about ten times the order of magnitude of the roughness of the surfaces.

**Figure 8:**
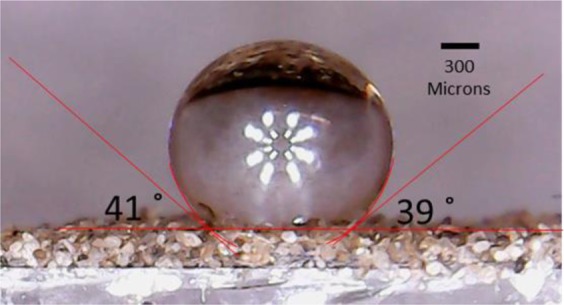
An example of measurement of apparent contact angle on hydrophobic rough surface.

All experiments are conducted on a vibration-free surface. A camera lens (MicroCapture, Lenovo Group Ltd.) with magnification of up to 1,000 times was employed to obtain images from which apparent contact angles were estimated. After the water droplet was placed on the hydrophobic surface, the camera lens is focused on the water droplet with lens surface maintained perfectly vertical to the horizon of the surface. After the first image is taken, the roughened surface with the droplet is rotated clockwise by 5° and another image is taken (after slight refocusing if required). For each rotation of the droplet, 72 images are taken. The rotation of the sample is repeated until a two full circles are completed. The rotations are done as quickly as possible (<5 s per rotation) to ensure that evaporation is small – the repeated images of the droplet and the absence of change of the angle when repeated demonstrate that evaporation of the droplet is negligible. An example of how apparent contact angles are determined is shown in Fig. 8. The repeated measurements given an observation error of ±2°.

After the first rotations are complete, the droplet is wicked away from the surface by using a paper towel (ensuring no physical contact of the towel and the roughened surface so as to make sure that no changes to the physical surface occurs). The dry weight of the towel is measured and then the wet towel weight is measured to determine the amount of water that is removed from the droplet by using a moisture analyzer (MS-70 Moisture Analyzer, A&D Company. Ltd). In all cases, a residual amount of water is left on the roughened surface. Thereafter, a second 5 µL DI water droplet was placed in the same location as the first droplet and the images are taken at 5° angle increments for two rotations (the syringe, center of rotator and substrate stage are fixed with respect to each other) as described above. After the images are taken, the water is again wicked away and the water amount removed is determined and a third droplet is placed on the roughened surface. Thereafter, the apparent contact angles are again measured as described above.

The first set of contact angles are measured at zero residual water content. From the mass balance of the droplet volumes and water removed, the residual amount of water remaining on the roughened surface can be determined. For each of the three hydrophobic surfaces, there are three data sets of contact angles both reflecting the contact angle heterogeneity around the water droplet and the impact of remaining water film on measurements.

The contact angle was also determined for a water droplet placed on a smooth hydrophobic silica surface to obtain the equilibrium contact angle.

## Results and Discussion

### Apparent contact angle

**Figure 9:**
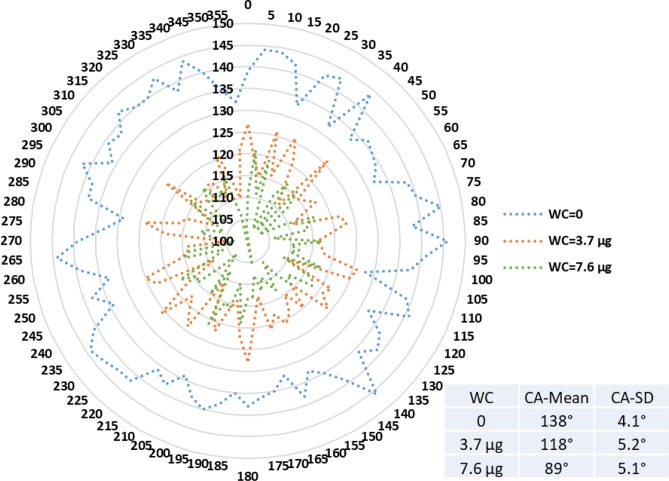
Contact angle distribution for hydrophobic sand surface (CA-Mean is the sample mean).

**Figure 10:**
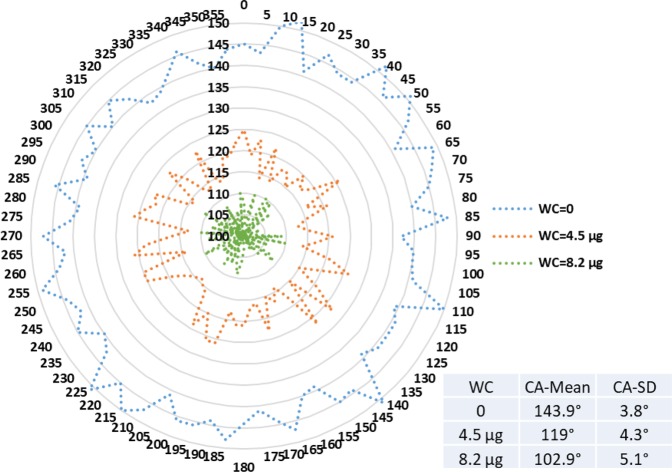
Contact angle distribution for hydrophobic glass bead surface (CA-Mean is the sample mean).

**Figure 11:**
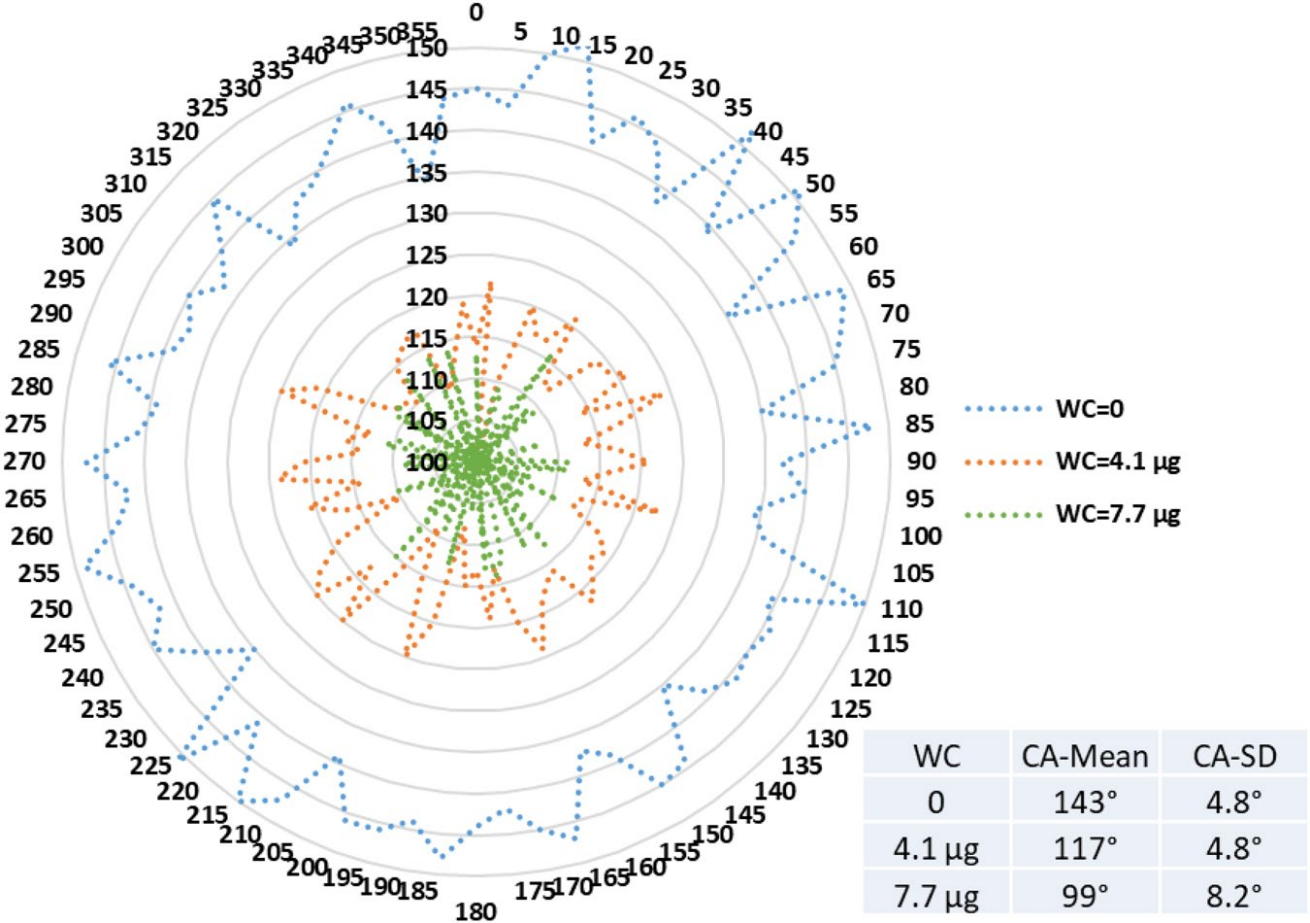
Contact angle distribution for hydrophobic mixed sand-bead surface (CA-Mean is the sample mean).

**Figure 12:**
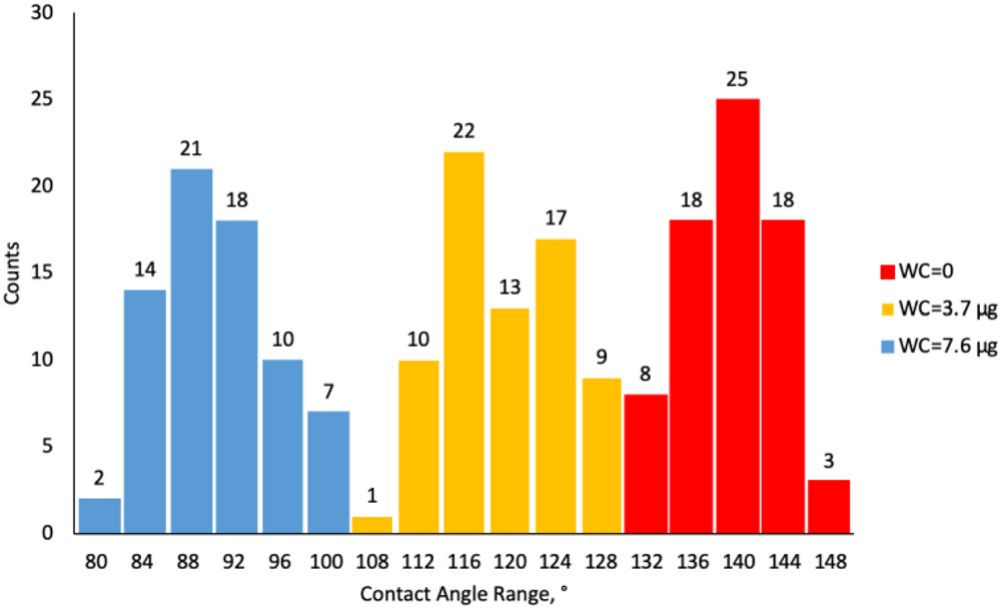
Histogram of contact angles for hydrophobic sand surface at different water content.

**Figure 13:**
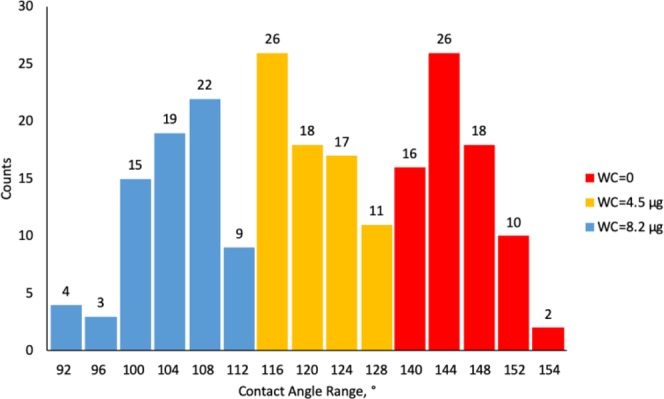
Histogram of contact angles for hydrophobic glass bead surface at different water content.

**Figure 14:**
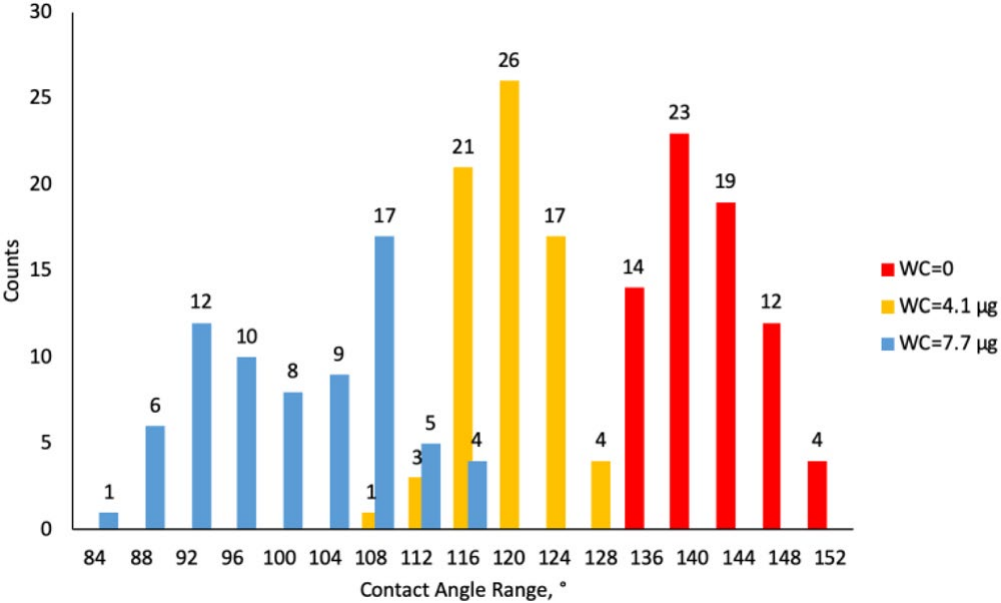
Histogram of contact angles for hydrophobic mixed sand-bead surface at different water content.

**Figure 15:**
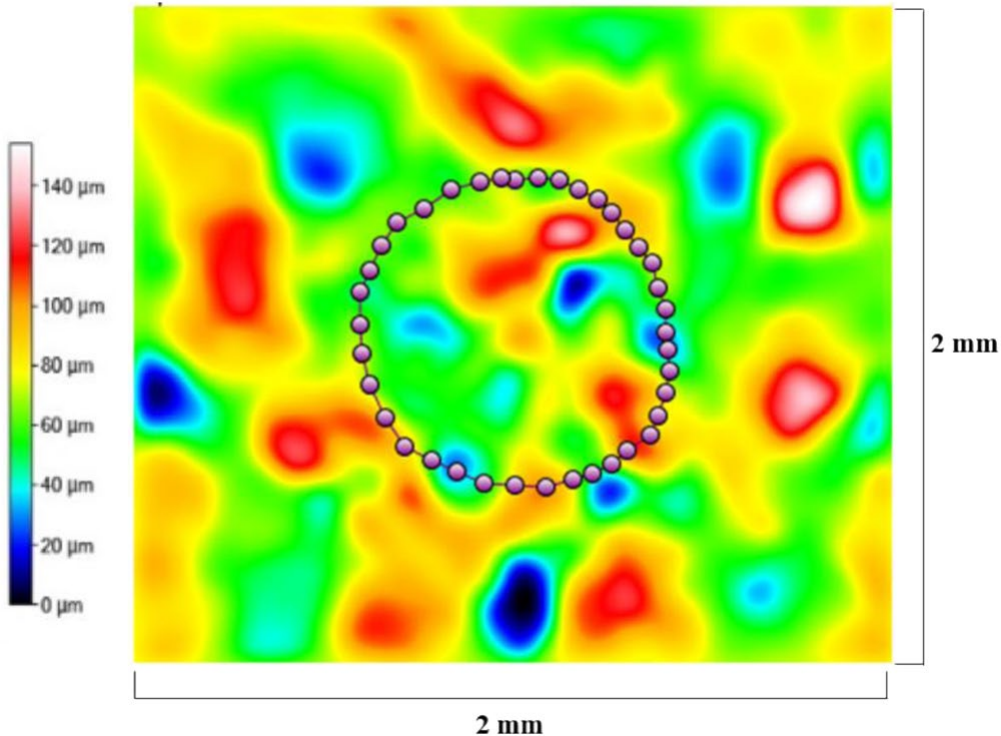
Contact line location for hydrophobic sand surface. The valley volume associated with this surface is equal to 3,976,000 μm^3^.

**Figure 16:**
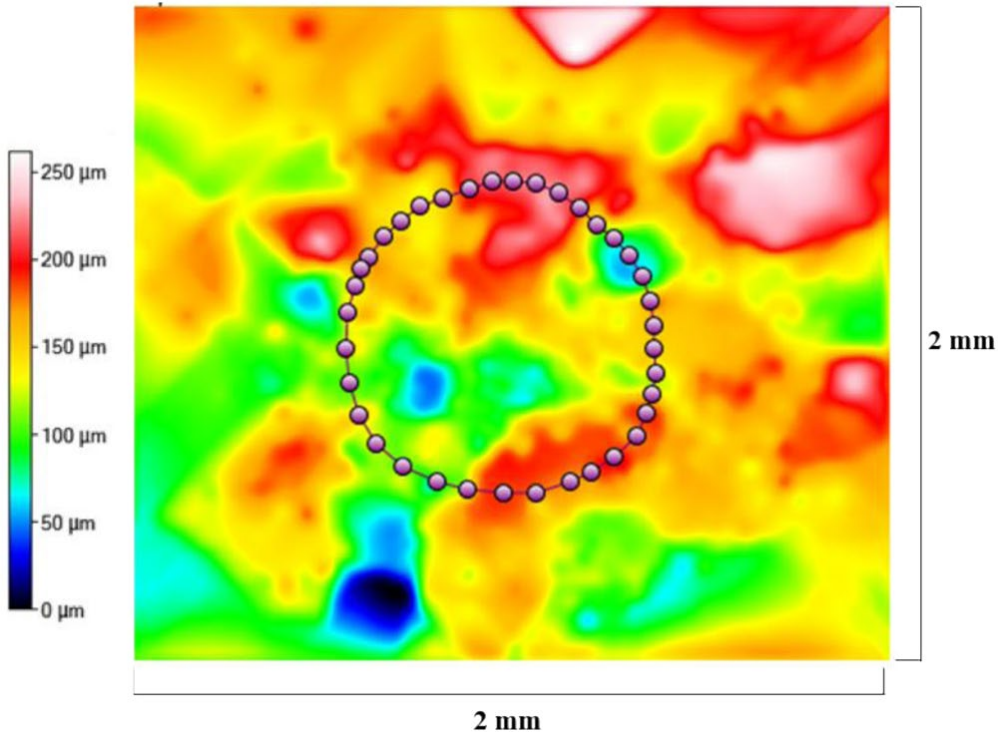
Contact line location for hydrophobic glass bead surface. The valley volume associated with this surface is equal to 11,230,000 μm^3^.

**Figure 17:**
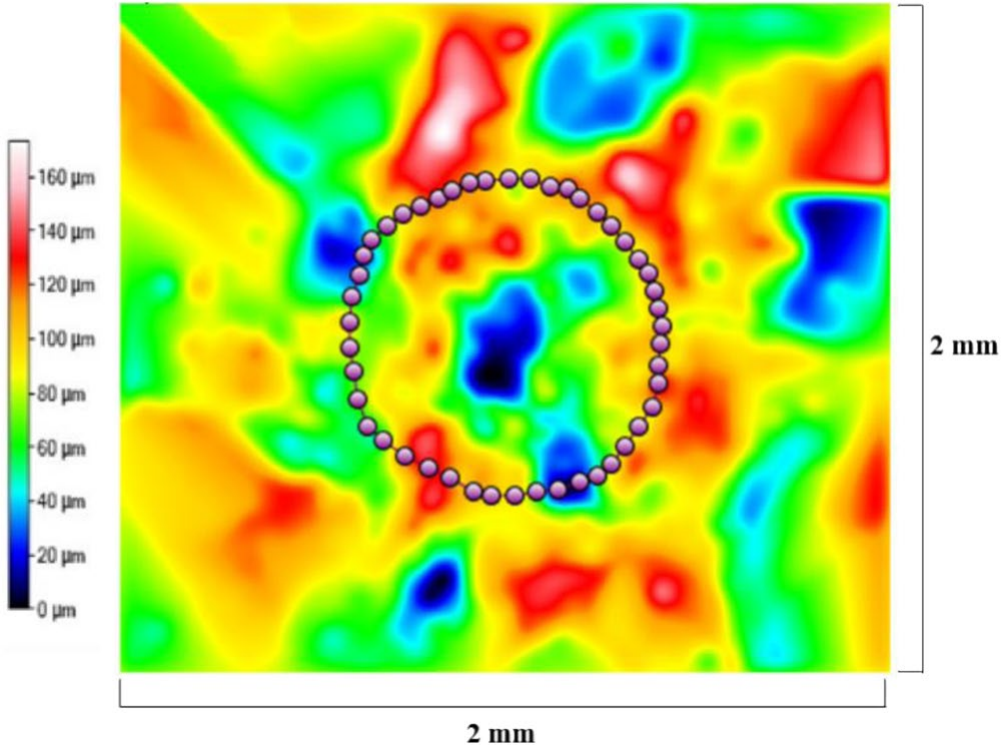
Contact line location for hydrophobic mixed sand-bead surface. The valley volume associated with this surface is equal to 8,946,000 μm^3^.

Figures 9–11 display radar charts of the contact angles around the water droplet from the top view at different residual water content of hydrophobic sand surface, hydrophobic glass bead surface and hydrophobic mixed sand-bead surface, respectively. Histograms of the contact angles at different residual water content of hydrophobic sand surface, hydrophobic glass bead surface and hydrophobic mixed sand-bead surface are shown in Figs. 12–14, respectively. Other statistical results of contact angle measurements at zero residual water content along with surface roughness are listed in Table 2. The results demonstrate that an increase of the roughness leads to an increase of the contact angle variability, so that for a severe rough surface, a single contact angle measurement or even the mean contact angle no longer reflects the real and complex contact line between liquid and solid. Additionally, the sand grain size variability and random arrangement of sand grains affects the effective average contact angle even when similar roughness is present. Hsieh *et al*.^26^ demonstrated experimentally that at nanoscale, for an originally hydrophobic surface, the increase of roughness contributes to the hydrophobicity of the surface with an increase of the contact angle. The observations in all experiments are at micron scale but show that the same trend occurs even at this larger scale. Even though the three different rough surfaces are all coated with hydrocarbon, there is an enhancement of the effective hydrophobicity as the roughness rises. As a reference, a smooth glass slide coated with the same material gives a uniform contact angle of 132° when a 5 µL of DI water droplet is placed on it. During the experiment, after the first water droplet is wicked away, there is a “butt stain” of water left on surface. An approximate contact area can be estimated from the mark by using Profilometer (Profilm3D, Filmetrics Inc.) software, and within the contact area, the volume of the ‘valleys’ as well as the ratio *R* given in Eqs. 2 and 3 can be estimated. Figures 15–17 show the calculated results and the top view of each hydrophobic surface. The dotted line in each figure represents the estimated contact line. During each experiment when the initial drop was placed on the surface, from the camera image, we observe intimate contact between droplet and solid surface indicating that a full Cassie-Baxter wetting state did not occur. The droplet trapped a small amount of air in the valleys and thus, it is also not in a Wenzel wetting state either. In Table 3, we list the apparent contact angles, ${\theta }_{A}$, calculated from Eqs. 2 and 3. Because it is difficult to estimate the volume of trapped air in the valleys, both *f* = 0.6 and 0.9 are used in Eq. 3. From the results in Table 3, the apparent contact angles from both Wenzel’s and Cassie-Baxter’s models are larger than that compared to the experimental data. As the surface roughness rises, the increasing value of *R* leads to a larger difference between calculated values and measured values. For the Cassie-Baxter model, the lower the value of *f*, the greater is the deviation of the model from the experimental data.

**Table 2:** Average apparent contact angles for the different rough surfaces.

Surface	Contact Angle (Mean)	Contact Angle (Median)	Contact Angle (Standard Deviation)
0	132°	132°	<0.1°
1	138°	138°	4.3°
2	143°	142°	4.8°
3	144°	144°	3.8°

**Table 3:** Apparent contact angles obtained from the different wetting models for initial drop placed on rough surface.

Surface	*R*	Wenzel Model	Cassie-Baxter Model, *f* = 0.6	Cassie-Baxter Model, *f* = 0.9	Measured Contact Angle (Mean of Samples measured)
1	1.13	139°	149°	141°	138°
2	1.22	145°	153°	147°	142°
3	1.34	154°	160°	155°	144°

In Figs. 9 to 14, the water content (WC) is the residual water content in micrograms, that is, the weight of the water remaining on surface valleys after the drop is wicked away. From Figs. 4 to 6, it is clear that the rough surfaces has its own hills and valleys, which when a residual water layer is left on the roughened surface, is similar to archipelago system with the surface peaks emerging as islands above the water layer. When the first water drop is placed on each surface, each surface demonstrates its hydrophobicity. Given the mass of residual water content, arithmetic mean height of surface, and groove volume within contact area, the average height of the remaining water film can be calculated. The results are listed in Table 4. With the increase of the water film filling the valleys, the apparent hydrophobicity decreases. If the residual water content is high enough, the surface shifts to a more effectively neutral surface than its original hydrophobic wettability. For the sand and mixed surfaces, when the residual water content reaches a similar value (7.6 $\mu g$ and 7.7 $\mu g$, respectively), the decrease in contact angle is more significant than that of the sand surface. The reason is due to the residual water spreading more evenly and widely for the less rough surface, which gives the newly placed water droplet a greater tendency to spread on the surface.

**Table 4:** Residual water amount on the rough surfaces.

Surface	Groove Volume, µL	Second Droplet		Third Droplet	
		Water Content, µL	Average Height of Remaining Water Film, µm	Water Content, µL	Average Height of Remaining Water Film, µm
1	0.004	0.0037	15.7	0.0076	32.2
2	0.009	0.0041	10.4	0.0077	19.5
3	0.011	0.0045	7.4	0.0082	13.5

Wenzel’s wetting is the case where the liquid completely fills the valleys of the rough surface. The prediction from Wenzel’s model listed in Table 3 suggest that the contact angle will enlarge due to the roughness of the surface. Given the wetted-surface droplet angles, the opposite trend is observed where the greater the amount of water trapped in the valleys, the lower is the average contact angle. This demonstrates a deviation from Wenzel’s model. In the Cassie-Baxter model, as the value of *f* decreases, the higher should be the contact angle. But this appears to be inconsistent with the experimental results obtained here as shown in Figs. 9 to 11 in the sense that the greater the residual water on the surface (lower *f*), the lower the contact angle. This deviation may stem from the fact that Cassie-Baxter’s model considers the air as a trapped phase, but in our case, the lower phase is water which has major positive impact on apparent hydrophilicity. One reason for the deviation of the models from the experimental results is that the droplet is more than ten times the length scale of the roughness; it has been shown in previous work that the droplet should be much greater (>500) than the scale of the roughness for the models to provide a good representation of the apparent contact angle^2,3,8^.

The ratio *R* represents the actual to projected area ratio is used in Eqs. 2 and 3 because the bulk liquid contact will affect the movement of the contact line. However, the contact angle is measured at the contact line which implies that an areal ratio of roughness may not be the appropriate scaling ratio. Rather, the line ratio of the actual wetting line path on the rough surface and the projected length of the contact line may be more appropriate and thus, $R \sim \sqrt{\frac{{A}_{rough}}{{A}_{projected}}}.$ This is a potential reason why both overestimate the apparent contact angle.

### Contact angle and roughness

For a macro-scaled rough surface, the measured contact angle varies over a large range. The results in Figs. 12 to 14 demonstrate that the distributions of contact angles changes from when the initial water drop is placed on the rough surface to subsequent drops placed on the wettened surface. When the initial drop is placed on the surface, for all three surfaces examined here, the contact angles generally follow a normal distribution. The distributions in Fig. 7 show that the larger the roughness, the greater is the width of the distribution of heights in the sample. This is similar to the results of the contact angle where the distribution obtained from the sand is narrower than that of the glass beads. For the wetted surfaces, the results show that the distributions of the contact angles become broader and that the distributions are more complex exhibiting greater skewness and multimodal distributions.

## Conclusions

For macroscale rough hydrophobic surfaces where roughness features are of order of tens to hundreds of microns, the results shows the same tendency as has been observed for nanoscale hydrophobic surfaces in that the increase of the roughness leads to higher apparent hydrophobicity. Around the periphery of a droplet deposited on a rough surface, the contact angle varies around the measured droplet generating an approximately normal distribution on a dry rough surface. This is similar to the distribution of the height of the roughness which suggests that the roughness distribution could be used to model the distribution of the contact angle; more work is needed to establish this. For a micron-scaled, dry rough surface, both Wenzel’s and Cassie-Baxter’s models tend to overestimate the apparent contact angle, but as the roughness decreases, both models are able to provide more accurate results. For a previously dry rough surface that has a residual water film on it, the apparent contact angle distribution is more complex without following a specific type of distribution model – skewness and multimodal distributions are observed. Although the dry surfaces show high hydrophobicity, an increase of the residual water content lowers the apparent contact angle shifting the surface towards a reduced apparent hydrophobicity. However, as long as the droplet has continuous liquid-solid contact, the surface still show apparent hydrophobicity even in presence of residual water content. The results suggest that the roughness of surfaces could be designed, even at the scale of tens to hundreds of microns, to enhance the hydrophobicity of the surface – this scale of roughness may be more practical than nano-manufactured roughened surfaces with respect to cost and application. However, such surfaces, after having been wetted, will have lower apparent contact angles than that of the originally dry surface. This implies that for designed surfaces, such surfaces should have high drainage capabilities to remove the wetting fluid so as to maintain their original hydrophobic characteristics.
